# Microbial responses to long-term warming differ across soil microenvironments

**DOI:** 10.1093/ismeco/ycae051

**Published:** 2024-04-06

**Authors:** Xiao Jun A Liu, Shun Han, Serita D Frey, Jerry M Melillo, Jizhong Zhou, Kristen M DeAngelis

**Affiliations:** Department of Microbiology, University of Massachusetts, Amherst, MA 01003, United States; Institute for Environmental Genomics and School of Biological Sciences, University of Oklahoma , Norman, OK 73019, United States; Institute for Environmental Genomics and School of Biological Sciences, University of Oklahoma , Norman, OK 73019, United States; Department of Natural Resources and the Environment, University of New Hampshire, Durham, NH 03824, United States; The Ecosystems Center, Marine Biological Laboratory, Woods Hole, MA 02543, United States; Institute for Environmental Genomics and School of Biological Sciences, University of Oklahoma , Norman, OK 73019, United States; Earth and Environmental Sciences, Lawrence Berkeley National Laboratory, Berkeley, CA 94720, United States; School of Civil Engineering and Environmental Sciences and School of Computer Science, University of Oklahoma, Norman, OK 73019, United States; Department of Microbiology, University of Massachusetts, Amherst, MA 01003, United States

**Keywords:** carbon storage and sequestration, bacterial necromass, substrate accessibility, biogeochemical cycles, soil aggregation, microbial evolution, organic matter decomposition, functional genomics, degradation enzymes, plant soil interactions

## Abstract

Soil carbon loss is likely to increase due to climate warming, but microbiomes and microenvironments may dampen this effect. In a 30-year warming experiment, physical protection within soil aggregates affected the thermal responses of soil microbiomes and carbon dynamics. In this study, we combined metagenomic analysis with physical characterization of soil aggregates to explore mechanisms by which microbial communities respond to climate warming across different soil microenvironments. Long-term warming decreased the relative abundances of genes involved in degrading labile compounds (e.g. cellulose), but increased those genes involved in degrading recalcitrant compounds (e.g. lignin) across aggregate sizes. These changes were observed in most phyla of bacteria, especially for *Acidobacteria*, *Actinobacteria*, *Bacteroidetes*, *Chloroflexi*, and *Planctomycetes*. Microbial community composition was considerably altered by warming, leading to declined diversity for bacteria and fungi but not for archaea. Microbial functional genes, diversity, and community composition differed between macroaggregates and microaggregates, indicating the essential role of physical protection in controlling microbial community dynamics. Our findings suggest that microbes have the capacity to employ various strategies to acclimate or adapt to climate change (e.g. warming, heat stress) by shifting functional gene abundances and community structures in varying microenvironments, as regulated by soil physical protection.

## Introduction

Microbes play a crucial role in soil organic matter (SOM) decomposition and have the potential to accelerate soil carbon loss to the atmosphere in response to climate warming [1–4]. Previous studies have demonstrated that warming is associated with increased abundances of functional genes involved in the degradation of organic matter with varying levels of recalcitrance [5–9] and enriched pathways related to cellulose degradation [10–12], but the effects on microbial abundances and community structure have been variable [13–17]. In soils, substrate availability is affected by physico-chemical protection mechanisms like adsorption, desorption, and aggregate turnover that decrease depolymerization and microbial decomposition [18], but the extent to which functional genes, metabolic pathways, and taxonomic groups vary with chronic warming, especially in different aggregate sizes, remains poorly studied.

Soil structure and mineralogy drive microbial community composition by affecting substrate availability and physical accessibility [19–24]. The presence of physical barriers within soil aggregates can protect SOM from decomposition by inducing microenvironmental constraints on decomposer movement and metabolism [25–28]. Different microbial groups also exhibited varying relative abundances between aggregates, with bacteria and fungi showing distinct patterns [29–31]. For instance, bacterial communities were found to be less diverse [22], while fungi were more diverse in macroaggregates than in microaggregates [32, 33], where the competition between the two groups was stronger [34, 35]. Furthermore, fungi may play a more dominant role in forming macroaggregates [36–39], although the ratio of fungi to bacteria can vary substantially [40]. Therefore, a fine-scale understanding of soil microbial community distribution is essential to comprehend how climate change impacts species interactions and metabolisms [41], and whether carbon persists in soils [42].

Previously we reported on two studies that explored how long-term warming at the Harvard Forest has affected microbes in different microenvironments [20, 21]. Soil samples from the control and heated plots were separated into macroaggregate (250–2000 μm) and microaggregate (<250 μm) fractions, and microbial carbon use efficiency (CUE) was measured with ^18^O enriched water (H_2_^18^O) in samples incubated at 15 or 25°C for 24 h. We found that warming reduced soil carbon and nitrogen concentrations, extracellular enzyme activities, microbial growth, respiration, and the temperature sensitivity (Q_10_) of CUE in macroaggregates [21]. To further explore how physical protection may inhibit SOM decomposition, we crushed aggregates to reduce physical protection and compared them to intact aggregates. We found that warming was associated with a smaller effect of physical protection for respiration but with a larger effect for biomass turnover rate in macroaggregates [20], suggesting that microbial functional traits vary across microenvironments with different physical protection, yet their responses to climate warming remain unclear.

In the new study we report here, we used metagenomics to examine how long-term warming affects the gene abundances and community structures of soil microbiomes associated with both macroaggregates and microaggregates. Because of their known differences in physical protection, our study aimed to provide insights into the mechanisms behind the differential effects of long-term warming on microbial functions and dynamics between macroaggregates and microaggregates. We posited that the effects of warming on gene abundances would be more pronounced in macroaggregates than in microaggregates. We further hypothesized that warming would increase the abundances of functional genes related to cell maintenance and degradation of complex substrates, but would reduce the abundances of genes responsible for degrading labile substrates, corresponding to the changes in abundances of oligotrophs and copiotrophs.

## Materials and methods

### Field site and sample processing

Samples were collected as previously described [20, 21] from the Harvard Forest long-term warming experiment (Pertersham, MA), where soils have been heated 5°C above ambient temperature since 1991 alongside control and disturbance control (instrumented but not heated) plots [43]. The soil for this study was collected in October 2017 from the mineral horizon (10 cm depth; *N* = 3), from the same batch of samples used for prior experiments [20, 21]. The soil was air-dried at 4°C until it reached a moisture level of about 10% to minimize any potential disturbance to the microbial communities during aggregate fractionation [20, 21, 44]. Microaggregates (<250 μm) and macroaggregates (250–2000 μm) were separated with a 250 μm sieve, weighed, and stored at −80°C for molecular analysis. To extract DNA from the soil aggregates, 0.5 g of each sample was weighed out and processed using the DNeasy PowerSoil Kit (Qiagen, Hilden, Germany). The extracted DNA was assessed for quality using the NanoDrop One (Thermo Scientific), quantified with the PicoGreen Kit (Quant-IT, Invitrogen), and treated with RNase A to avoid potential RNA contamination.

### Processing of metagenomic data

Shotgun metagenome library preparation, sequencing, assembly, and annotation were performed at the Joint Genome Institute (JGI) following standard protocols and pipelines [45]. Soil DNA samples were sequenced at JGI using a NovaSeq 6000 System (2 × 150 bp; Illumina). The samples had an average guanine-cytosine (GC) content of 62.1%, reads of 3 942 584 112, and 94.9% of reads ≥Q30 (BBTools, v38.59; Supplementary Table S1). The filtered quality reads were assembled with metaSPAdes (version 3.13.0) using different k-mer lengths [46, 47]. On average, 25.3 Gb of MG sequence was obtained per soil sample.

To annotate the functional profiles of the metagenomes, we employed the Integrated Microbial Genomes Annotation Pipeline (v.5.0), which assigned protein-coding genes to Clusters of Orthologous Groups (COGs), Enzyme Commission (EC) numbers, and KEGG Orthology terms [45, 48, 49]. Protein-coding genes were compared against high-quality genomes using USEARCH 6.0.294 to assign EC numbers [50], which were then mapped to the Carbohydrate-Active Enzymes database (CAZy) for taxonomic analysis of all functions [51]. Phylogenetic distributions were determined using the best BLAST hits against a non-redundant protein database derived from high-quality genomes based on the Integrated Microbial Genomes (IMG) platform. To analyze taxonomy, COG taxon-specific functions were filtered to include only hits with >30% identities at the phylum level. To account for differences in metagenome size, all functional abundance matrices were normalized to hits per million reads [52].

### Taxonomic annotation of genes in metagenome

To assess the taxonomic composition of a genome sample, the distribution of best BLAST hits of protein-coding genes was conducted in the dataset. Instead of e-value filtering, IMG utilizes percent identity filtering for best hits to account for the variation in alignment length between query and target proteins. Percent identity ranges used in IMG roughly correspond to the average amino acid identity found between genomes from the same genus (90 + % range), the same family or order (60%–89%), and same class or phylum (30%–59%). Additionally, IMG provides links to COG functional category information for genes with 30%, 60%, and 90% hits. Here we selected the 30% identity to investigate microbial taxonomic responses at the phylum/class level and classified the best hits into two COG functional categories: metabolism (amino acid transport, carbohydrate transport, energy production, lipid transport, coenzyme transport, inorganic ion transport, nucleotide transport, secondary metabolites biosynthesis) and cellular processes (cell wall biogenesis, defense mechanisms, signal transduction, posttranslational chaperones, cell division, cell motility, extracellular structures, and intracellular trafficking).

### CAZy degradation genes

We employed the CAZy database, which relies on functionally related domains of enzymes that break down glycosidic bonds, to classify the enzyme genes [53]. Our study focused on four classes of enzymes: auxiliary activities, carbohydrate esterases (CE), glycoside hydrolases (GH), and polysaccharide lyases (PL). These enzymes were assigned to substrates such as hemicellulose, starch, cellulose, chitin, pectin, and lignin, as listed in Supplementary Table S2. Based on the origins, genes associated with peptidoglycan and chitin degradation were grouped for bacterial and fungal biomass degradation, while hemicellulose, cellulose, and lignin for plant biomass degradation.

### Taxonomic analysis

We employed metagenomic data to evaluate the abundance of bacterial and fungal communities in control and heated plots. We utilized Kraken2 (v. 2.0.9) to assign taxonomic groups with the National Center for Biotechnology Information (NCBI) taxonomy, which included bacteria, fungi, and archaea. The taxon-by-sample matrices were normalized based on the total number of reads per sample to mitigate the impact of uneven sequencing depth. The relative abundances of phyla or classes were calculated using the abundances of sequences that matched archaea, bacteria, or fungi [54].

### Statistical analyses

To minimize the effect of variation in sequencing depth among samples, functional genes and relative abundances of taxa were normalized to hits per million reads. We conducted non-metric multidimensional scaling using Bray–Curtis dissimilarities based on square root transformed abundances of functional genes or relative abundances of bacterial or fungal communities. Shannon’s diversity was used to measure community evenness and richness. To test for differences in bacterial communities, we performed Permutational Multivariate Analysis of Variance and homogeneity of variances using the functions adonis and betadisper in R [8]. All sequencing data were analyzed based on three biological replicates per sample. *P* values for the relative abundances of taxa and functional genes were calculated using the two-tailed *t*-test. All statistical analyses were performed with R [55]. Due to the small sample size, a *P* value of ≤.10 was considered statistically significant for the tests [10, 51, 56].

## Results and discussion

Our prior findings show that long-term warming reduced soil carbon and nitrogen concentrations, activities of hydrolytic and oxidative enzymes, microbial growth, respiration, and Q_10_ of microbial CUE in macroaggregates but not in microaggregates [20, 21]. Besides, warming showed a larger effect on microbial biomass turnover but smaller effect on respiration in macroaggregates than in microaggregates. To understand how microbial functional traits vary in their responses to long-term warming across aggregates, here we conducted metagenomic analysis on the same microaggregates and macroaggregates from the heated and control soils. Our current study shows the interactive effects of warming and physical protection on microbiome functions, where soil aggregates drive microbial community composition and functional genes by influencing the substrate availability and physical accessibility.

### Warming effects on functional genes are partly regulated by microenvironments

**Figure 1:**
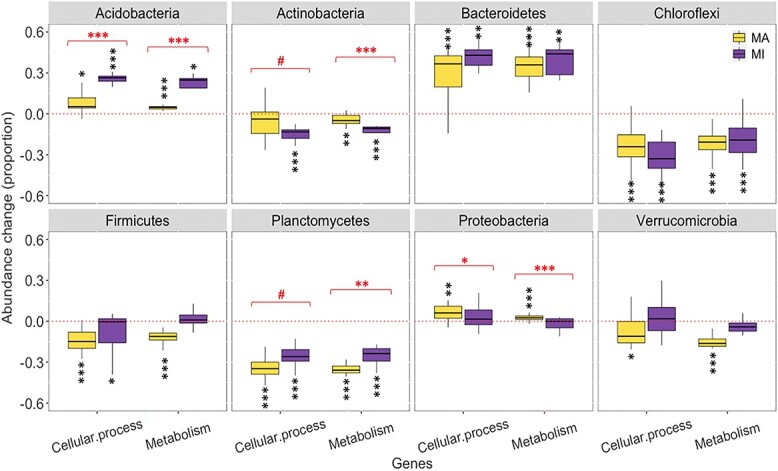
Changes of microbial functional genes associated with metabolism and cellular processes in different aggregates under long-term warming; MA and MI are macroaggregates and microaggregates (250–2000; <250 μm); black symbols indicate significant effect sizes of warming ([heated-control]/control), while red symbols indicate significant differences between MA and MI (^#^, ^*^, ^**^, ^***^ at *P* < .10, .05, .01, and .001).

Our findings partly support the hypothesis that long-term warming would increase the abundances of genes responsible for cellular processes and metabolism. Specifically, warming consistently increased the gene abundances of *Acidobacteria*, *Bacteroidetes*, *Crenarchaeota*, *Euryarchaeota*, *Gemmatimonadetes*, *Proteobacteria*, and *Ascomycota*, while reduced the gene abundances for *Actinobacteria*, *Armatimonadetes*, *Chloroflexi*, *Firmicutes*, *Planctomycetes*, *Basidiomycota*, and *Zoopagomycota* in both macroaggregates and microaggregates (Fig. 1, Supplementary Figs S1–S3). Compared to macroaggregates, gene abundances in microaggregates increased more for *Acidobacteria* and *Ascomycota* with warming but less for *Proteobacteria*, and decreased more for *Actinobacteria* but less for *Planctomycetes*. These findings suggest that warming effects on functional genes vary depending on the specific microbial communities that are partly impacted by aggregates [8, 57], highlighting the need for further research to understand the physical protection associated mechanisms in driving microbial responses to climate warming.

**Figure 2:**
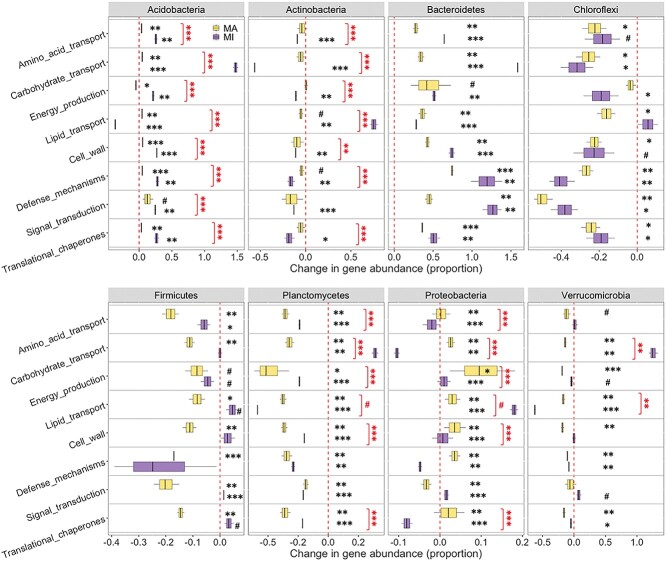
Changes of microbial functional genes in different aggregates under long-term warming; MA and MI are macroaggregates and microaggregates (250–2000; <250 μm); black symbols indicate significant effect sizes of warming ([heated-control]/control), while red symbols indicate significant differences between MA and MI (^#^, ^*^, ^**^, ^***^ at *P* < .10, .05, .01, and .001).

Our analysis of metabolic pathways revealed that the effects of long-term warming on gene abundances varied depending on individual functional traits and phyla. Under warming for both macroaggregates and microaggregates, abundances of genes associated with (1) amino acid transport increased for *Acidobacteria*, *Bacteroidetes*, *Gemmatimonadetes*, *Thaumarchaeota*, while decreased for *Chloroflexi*, *Firmicutes*, *Planctomycetes*, *Spirochaetes*, and most fungal phyla; (2) carbohydrate transport increased for *Acidobacteria* and *Bacteroidetes*, while decreased for *Chloroflexi*, *Deinococcus-Thermus*, *Zoopagomycota*; (3) lipid transport increased for *Bacteroidetes*, *Proteobacteria*, *Mucoromycota*, while decreased for *Armatimonadetes*, *Crenarchaeota*, *Cyanobacteria*, *Planctomycetes*, *Verrucomicrobia*, and *Basidiomycota* (Fig. 2, Supplementary Figs S4–S8). Our results, depending on specific phyla, are partly supported by previous studies that have reported increases in abundances of genes associated with lipids and polysaccharides metabolisms under chronic warming, and an increase in genes associated with carbohydrate metabolism in permafrost systems [12, 58]. These findings suggest that long-term warming may have selected microbial species and genes that can either adapt or resist to heat associated stress conditions [59]. Thus, depending on their genetic and physiological states in variable microenvironments, microbes likely respond in different ways [60, 61] to climate warming [62].

Our analysis of gene abundances associated with cellular processes revealed that long-term warming had consistent effects on certain functional traits and phyla, while had variable effects on the others. Specifically, warming consistently increased the gene abundances associated with cell wall biogenesis, defense, signal transduction, and transcriptional chaperones for *Acidobacteria*, *Bacteroidetes*, and *Gemmatimonadetes* in both macroaggregates and microaggregates (Fig. 2, Supplementary Figs S4–S8). However, warming reduced these gene abundances for *Actinobacteria*, *Armatimonadetes*, *Chloroflexi*, *Firmicutes*, and *Planctomycetes*. These findings highlight the inconsistent responses of functional traits associated with different phyla to environmental stress [63–65], and may allow for some predictive insights in feedbacks of soil carbon cycling to climate change.

### Warming effects on carbon degradation genes are independent of soil microenvironments

**Figure 3:**
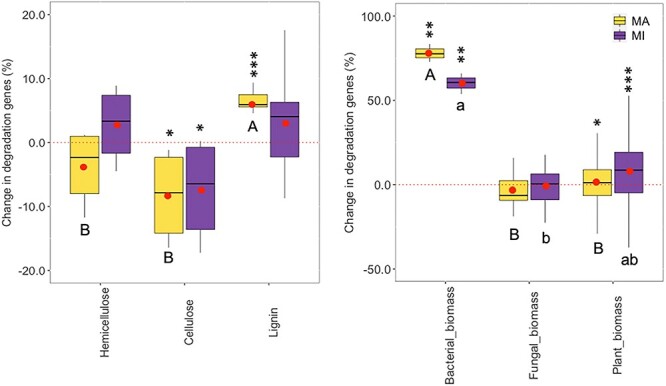
Changes of microbial functional genes associated with carbon degradation in different aggregates under long-term warming; MA and MI are macroaggregates and microaggregates (250–2000; <250 μm); black symbols indicate significant effect sizes of warming ([heated-control]/control ×100%) (^*^, ^**^, ^***^ at *P* < .05, .01, and .001); different uppercase and lowercase letters indicate differences within macroaggregates or microaggregates.

Our hypothesis was that chronic warming would lead to an increase in genes associated with the degradation of complex substrates and a decrease in genes associated with the degradation of labile substrates, consistent with previous observations of microbial responses to long-term warming [2, 66]. We found that warming increased the abundance of genes responsible for the degradation of lignin and reduced genes associated with cellulose degradation (Fig. 3), consistent with observations of decreased relative abundances of lignin in the heated soils [67]. A short-term warming reduced the genes associated with cellulose degradation but showed little effect on genes associated with degradation of hemicellulose and lignin [68]. However, we observed variable warming effects on the genes associated with hemicellulose degradation, with some genes showed either increased (CE4, GH10, PL5) or decreased abundances (GH38, PL8, PL12) in both macroaggregates and microaggregates (Supplementary Figs S9 and S10). Our previous work found that warming reduced potential enzyme activities for degradation of labile carbon but not for degradation of recalcitrant carbon [21], indicating that gene abundances may not necessarily reflect microbial potential functions. Chronic warming was reported to increase relative abundances of genes associated with degradation of labile carbon, but showed inconsistent effects on genes associated with complex carbon degradation in grasslands [8, 10, 11]. By contrast, short-term warming increased the abundances of functional genes involved in the degradation of labile and recalcitrant carbon in a permafrost system [7]. Our findings support the shift away from cellulose toward lignin as a source of substrates for soil heterotrophs based on declined nutrients [2, 6], though the capacity of microbes in degrading cellulose may increase with chronic warming [51].

Long-term warming had distinctive effects on the relative abundances of genes associated with degradation of bacterial, fungal, and plant biomass (Fig. 3), suggesting that chronic warming may have accelerated microbial decomposition of necromass under substrate- or nutrient-limited conditions [69]. However, in the long-term, both bacteria and fungi may still play critical roles in enhancing soil organic carbon (SOC) sequestration [32]. Our study provides further insight into the complex and dynamic responses of soil microbial communities to chronic warming and highlights the need for continued research to better understand these processes in different microenvironments with implications for soil carbon cycling.

### Warming effects on microbial community dynamics are influenced by microenvironments

**Figure 4:**
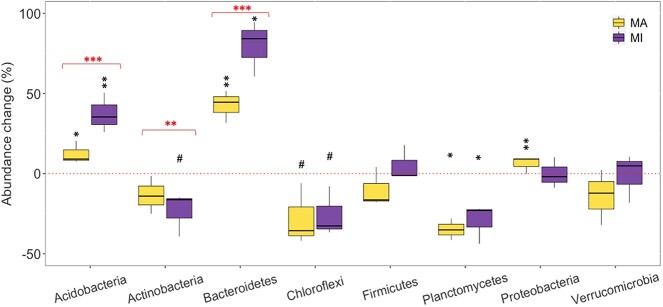
Changes of bacterial relative abundances in different aggregates over long-term warming; MA and MI are macroaggregates and microaggregates (250–2000; <250 μm); black symbols indicate significant effect sizes of warming ([heated-control]/control×100%)), while red symbols indicate significant differences between MA and MI (^#^, ^*^, ^**^, ^***^ at *P* < .10, .05, .01, and .001).

We hypothesized that relative abundances of copiotrophs would decrease, while oligotrophs increase after long-term warming. Our hypothesis was partly supported that the relative abundances of some copiotrophs (*Actinobacteria*) decreased and of some oligotrophs (*Acidobacteria*, *Euryarchaeota*, *Nitrospirae*) increased with warming (Fig. 4, Supplementary Figs S11–S14). However, warming also increased the relative abundances of other copiotrophs (*Bacteroidetes*, *β-/γ- Proteobacteria*, *Crenarchaeota*, *Gemmatimonadetes*) and reduced those of other oligotrophs (*Chloroflexi, Planctomycetes*). These findings suggest the difficulty in generalizing microbial life strategies [61], such as either an increase [14, 70, 71] or a decrease [72] in relative abundances of copiotrophs and oligotrophs with warming. Warming tended to reduce the relative abundances of most fungi (*Basidiomycota*, *Chytridiomycota*, *Zoopagomycota* except *Ascomycota*), suggesting that fungi are less competitive than bacteria under warming [73]. Yet, heat stress has been reported to increase the relative abundances of *Gemmatimonadetes*, *Verrucomicrobia*, *and Basidiomycota*, but to reduce the relative abundances of *Acidobacteria*, *Ascomycota*, *Firmicutes*, and *Myxococcota* in a cropping ecosystem [74]. Our findings suggest that even with decreased nutrients [2, 20, 21], oligotrophs and copiotrophs may occupy niches with adaptive traits to enhance their ability to outcompete other microbes, supporting microbial life history trade-offs between competition and resource acquisition under warming [9, 75–77]. Alternatively, warming may act as a filtering factor to impose positive or negative selection on spore-forming (e.g. *Actinobacteria, Firmicutes*) or non-spore-forming microbes [72]. Our research reinforces that the copiotroph–oligotroph classification may not accurately represent microbial thermal responses, particularly in different microenvironments [21, 23, 28, 78].

**Figure 5:**
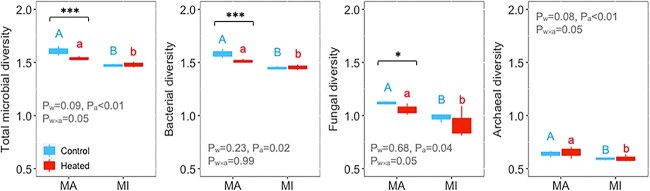
Microbial diversity as affected by aggregate size in response to long-term warming (different letters indicate differences between macroaggregates and microaggregates in control (blue) or heated plots (red); *P* values for diversity were obtained from two-way ANOVA (^*^ and ^*^^*^^*^ at P<.05 and .001).

**Figure 6:**
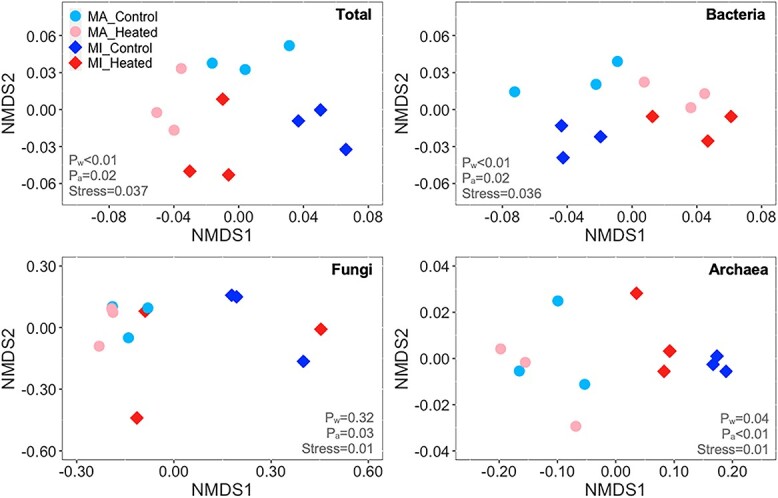
Community composition of different microbial groups as affected by aggregate size in response to long-term warming; *P* values for community composition were obtained from Permanova (Adonis) test; dispersion test (betadisper) showed no significant treatment effects.

We also hypothesized that chronic warming would reduce microbial diversity and alter community composition. As expected, long-term warming decreased the diversity of bacteria and fungi, but only in macroaggregates (Fig. 5). Microbial community composition shifted with warming and varied between macroaggregates and microaggregates (Fig. 6). These findings are consistent with previous studies that warming has reduced microbial diversity and altered community composition in various ecosystems [16, 33, 71]. However, some studies reported little change of microbial diversity or community composition in response to warming [2, 8, 15, 79]. These different responses of microbial communities to warming indicate complex interactions among soil substrate availability, community structure, and other abiotic factors [69].

### Aggregate physical protection regulates functional genes and community dynamics

Our initial hypothesis was that warming would have a greater impact on functional gene abundances in macroaggregates compared to microaggregates, but our findings show that warming effects were highly phylum-dependent (Figs 1 and 2). Compared to microaggregates, the warming effects on functional genes in macroaggregates were greater for *Planctomycetes* and *Proteobacteria* but smaller for *Acidobacteria* and *Actinobacteria* (Figs 1 and 2, Supplementary Figs S1–S8). Different warming effects on the gene abundances associated with cell motility genes were observed for *Armatimonadetes*, *Cyanobacteria*, *Deinococcus-Thermus*, *Gemmatimonadetes*, *Proteobacteria*, and *Basidiomycota* (Supplementary Figs S4–S8). Gene abundances of most functional traits increased with warming for some oligotrophic and copiotrophic microbes (*Acidobacteria*, *Bacteroidetes*, *Euryarchaeota*, *Gemmatimonadetes*, *Proteobacteria*), but decreased for others (Supplementary Tables S3–S5). Nevertheless, the warming effects varied significantly between macroaggregates and microaggregates, suggesting that the responses of microbial communities to warming may be different in moisture and nutrient availability among aggregates [80, 81]. The observed differences in gene abundances also suggest that even closely related microbial species may exhibit varying abilities with warming, underscoring complicated interactions between microbial communities and the microenvironments [82].

Warming effects on the relative abundances of genes degrading different substrates varied between aggregate sizes (Fig. 3, Supplementary Figs S9 and S10). For instance, warming increased the relative abundances of genes degrading hemicellulose, cellulose, lignin, and pectin in some enzyme families, but reduced the relative abundances of genes degrading hemicellulose and pectin in other families. Compared to microaggregates, the relative abundances were smaller for genes degrading cellulose, hemicellulose (e.g. CE4, GH38), and pectin in macroaggregates. Moreover, the warming effects on abundances of genes of degrading microbial biomass were stronger than those degrading plant biomass, especially in the macroaggregates, suggesting that microenvironment-controlled physical protection is an important determinant of microbial necromass in forming new SOM and increasing soil carbon storage (i.e. sequestration). These findings are also consistent with previous studies that the decomposition of different substrates is regulated by different microbial populations [83, 84]. The variable responses of microbes to warming could be due to differences in the physical and chemical properties between aggregates, which affect the availability of different substrates and the composition of microbial communities [54, 85].

We hypothesized that warming would have a greater impact on the relative abundances of microbial communities in macroaggregates compared to microaggregates. However, warming effects on the relative abundances of most phyla were smaller in macroaggregates than in microaggregates, suggesting that microbial functional differences can be largely attributed to different resource and moisture availability in microhabitats under warming [21, 86], rather than to the presence or absence of particular taxonomic groups [29]. Another possibility is that microbial communities might be associated with different predation pressures on populations between aggregates [31]. With warming, the relative abundances of some oligotrophs (*Armatimonadetes*, *Cyanobacteria*, *Deinococcus-Thermus*, and *δ-Proteobacteria*) increased in macroaggregates but decreased in microaggregates, suggesting that oligotrophs are more adapted in low-nutrient environments [21, 61, 87, 88]. The greater loss of SOC and nitrogen in macroaggregates and changes in relative abundances and functional genes under warming [20, 21] probably influenced microbial habitats and niches [42], indicating the potential influence of microbial communities on soil carbon and nitrogen cycling over climate change [89, 90].

Bacterial and fungal diversity was greater in macroaggregates than in microaggregates, with the bacterial diversity being greater than the fungal diversity, indicating consistent microbial responses at the domain level (Fig. 5). This finding is in accordance with previous research that microbial communities were associated with different soil aggregate fractions, highlighting the importance of the spatial distribution of bacterial and fungal communities [30, 33]. For example, bacterial diversity was smaller, while fungal diversity was greater in macroaggregates than in microaggregates [23, 91]. These results imply that biotic interactions within microenvironments could play important roles in regulating changes in biodiversity in response to climate warming.

We found distinct community composition between macroaggregates and microaggregates, indicating that the varying accessibility of nutrients [20, 21] may have induced the assembly of diverse communities [22]. For instance, different soil carbon quality has been suggested to cause the differentiation of microbial communities between free and occluded microaggregates [22]. However, bacterial and fungal community structures were similar across soil aggregates in agricultural systems [30]. These findings suggest that microbial communities might be susceptible to the effects of long-term climate warming, depending on different microenvironments and the associated physical protection.

## Conclusion

Our study suggests that microbial communities in different soil microenvironments (e.g. macroaggregates, microaggregates) could play distinct, independent, yet crucial roles in regulating soil carbon feedback responses to climate change. The reductions of microbial diversity, shifts in community composition, and variable changes in functional and taxonomic abundances resulting from warming, particularly in macroaggregates, suggest the significant potential of microbiomes to reduce soil carbon loss and enhance soil carbon storage (i.e., sequestration), depending on whether the dominant communities have declined or increased abundances of carbon degradation genes. A deeper understanding of the underlying microbial mechanisms and functions, substrate accessibility and availability, as well as the associated physical protection in the extremely heterogeneous soil environment could foster a better understanding of the larger-scale ecosystem responses. This represents a promising and critical frontier for improving climate-resilient models and managing soil microbiomes (e.g. improve soil health and develop sustainable agricultural and natural ecosystems), to mitigate the negative effects of climate change.

## Supplementary Material

Supplementary-materials_v12_ycae051

## Data Availability

Raw sequencing data were deposited at the JGI IMG platform (#503736). Other data supporting the findings of this study are available at the Harvard Forest Data Archive, consistent with the NSF LTER and Harvard Forest data policies [92].
