# Supplementary material for: Microbial responses to long-term warming differ across soil microenvironments
Source: ISME Commun. 2024 Apr 6;4(1):ycae051. doi: 10.1093/ismeco/ycae051 (PMC11065356; doi:10.1093/ismeco/ycae051)
Supplement: Supplementary-materials_v12_ycae051 [file supplementary-materials_v12_ycae051.docx]

# Microbial responses to long-term warming differ across soil microenvironments

Xiao Jun A. Liu^1,2,^*, Shun Han^2^, Serita D. Frey^3^, Jerry M. Melillo^4^, Jizhong Zhou^2,5,6^, Kristen M. DeAngelis^1,^*

^1^Department of Microbiology, University of Massachusetts, Amherst, MA 01003, United States. ^2^Institute for Environmental Genomics and School of Biological Sciences, University of Oklahoma, Norman, OK 73019, United States. ^3^Department of Natural Resources and the Environment, University of New Hampshire, Durham, NH 03824, United States. ^4^Ecosystems Center, Marine Biological Laboratory, Woods Hole, MA 02543, United States. ^5^Earth and Environmental Sciences, Lawrence Berkeley National Laboratory, Berkeley, CA 94720, United States. ^6^School of Civil Engineering and Environmental Sciences and School of Computer Science, University of Oklahoma, Norman, OK 73019, United States.

*Corresponding authors: Xiao Jun A. Liu, Institute for Environmental Genomics, University of Oklahoma, 101 David L Boren Blvd, Norman, OK 73019, United States. Email: [xj.allen.liu@gmail.com](mailto:xj.allen.liu@gmail.com) and Kristen M. DeAngelis, Department of Microbiology, University of Massachusetts, 639 N Pleasant Street, Amherst, MA 01003, United States. Email: [deangelis@microbio.umass.edu](mailto:deangelis@microbio.umass.edu)

| **Table S1.** Metagenomic data for soil samples at the chronic warming site at Harvard Forest, MA, United States. | | | | | | | |
| --- | --- | --- | --- | --- | --- | --- | --- |
| **Warm** | **Agg** | **Size (Gb)** | **Raw reads (million)** | **Filtered reads (million)** | **GC%** | **Contig count (million)** | **Reads mapped to contigs (%)** |
| Control | MA | 29.4 (1.7) | 396 (22) | 393 (21) | 62.3 (0.2) | 6.1 (0.3) | 67.3 (1.8) |
| Control | MI | 23.7 (1.4) | 342 (10) | 340 (10) | 62.6 (0.1) | 5.1 (0.3) | 63.9 (0.5) |
| Heated | MA | 24.6 (0.9) | 412 (38) | 407 (38) | 61.7 (0.0) | 6.3 (0.7) | 67.8 (1.6) |
| Heated | MI | 23.3 (0.4) | 359 (16) | 357 (16) | 61.8 (0.1) | 5.2 (0.2) | 66.7 (0.5) |
| Note: Warm = treatments for heated and ambient soils; Agg = macroaggregates (MA, 250-2000 µm) and microaggregates (<250 µm). Mean values from three field replicates, and standard error of mean are shown within parentheses. Raw reads were filtered by Q30 on the IMG platform from JGI. | | | | | | | |

| **Table S2.** List of main CAZy families encoding the enzyme activities involved in degradation of compounds from plant and microbial biomass. | | |
| --- | --- | --- |
| **Compound** | **Enzyme** | **CAZy family** |
| Starch | starch oxidase, monooxygenase, lytic polysaccharide monooxygenase, glucoamylase, glucodextranase. | AA13, GH15, GH65. |
| Hemicellulose | α-galactosidase, β-galactosidase, β-glucuronidase, endoxylanase, endoglucanase, xyloglucanase, β-xylosidase, α-L-arabinofuranosidase, acetyl xylan esterase, β-mannanase, α-N-acetylgalactosaminidase, α-glucuronidase, exo-α-L-1,5-arabinanase, α-L-fucosidase. | GH2-4, GH10, GH11, GH16, GH26, GH27, GH29-31, GH35, GH36, GH39, GH42-44, GH51, GH52, GH54, GH62, GH67, GH93, GH95, GH115, GH120, GH127, GH131, CE1-7, CE12, CE15, CE16. |
| Cellulose | β-glucosidase, endoglucanase, cellobiohydrolase, lytic polysaccharide monooxygenase. | GH1, GH5-9, GH12, GH45, GH48, GH74, GH94, GH116, AA9, AA10. |
| Pectin | polygalacturonase, xylogalacturonan hydrolase, pectin lyase, pectate lyase, oligogalacturonate lyase, pectin acetylesterase. | GH28, GH78, PL1, PL3, PL10, PL22, CE12, CE13. |
| Lignin | laccase, peroxidase, oxidase, 1,4-benzoquinone reductase, ferroxidase. | AA1, AA2, AA3, AA4, AA6. |
| Chitin | chitinase, N-acetyl β-glucosaminidase, lytic polysaccharide monooxygenase. | AA11, GH16, GH18-20, GH23, GH73, GH76, CE9. |
| Citations: [1–6] | | |

| **Table S3.** Responses of microbial functional genes to long-term warming in different soil aggregates. | | | | | | | | | | | | |
| --- | --- | --- | --- | --- | --- | --- | --- | --- | --- | --- | --- | --- |
| **Process** | **Function** | **Acidobacteria** | |  | **Actinobacteria** | |  | **Planctomycetes** | |  | **Proteobacteria** | |
|  |  | **MA** | **MI** |  | **MA** | **MI** |  | **MA** | **MI** |  | **MA** | **MI** |
| Cellular process |  | **B** | **A** |  | **B** | **A** |  | **A** | **B** |  | **A** | **B** |
|  |  |  |  |  |  |  |  |  |  |  |  |  |
|  | Cell wall | **B** | **A** |  | **B** | **A** |  | **A** | **B** |  | **A** | **B** |
|  | Defense mechanisms | **B** | **A** |  | **B** | **A** |  |  |  |  | **A** | **B** |
|  | Signal transduction | **B** | **A** |  |  |  |  |  |  |  | **B** | **A** |
|  | Translational chaperones | **B** | **A** |  | **B** | **A** |  | **A** | **B** |  | **A** | **B** |
|  | Cell division |  |  |  |  |  |  |  |  |  |  |  |
|  | Cell motility |  |  |  |  |  |  |  |  |  |  |  |
|  | Extracellular structures |  |  |  |  |  |  | **A** | **B** |  |  |  |
|  | Intracellular trafficking | **B** | **A** |  | **A** | **B** |  | **A** | **B** |  |  |  |
|  |  |  |  |  |  |  |  |  |  |  |  |  |
| Metabolism |  | **B** | **A** |  | **B** | **A** |  | **A** | **B** |  | **A** | **B** |
|  |  |  |  |  |  |  |  |  |  |  |  |  |
|  | Amino acid transport | **B** | **A** |  | **B** | **A** |  | **A** | **B** |  | **B** | **A** |
|  | Carbohydrate transport | **B** | **A** |  | **B** | **A** |  | **B** | **A** |  | **A** | **B** |
|  | Energy production | **B** | **A** |  | **B** | **A** |  | **A** | **B** |  | **A** | **B** |
|  | Lipid transport | **B** | **A** |  | **B** | **A** |  | **B** | **A** |  | **B** | **A** |
|  | Coenzyme transport | **B** | **A** |  | **B** | **A** |  | **A** | **B** |  |  |  |
|  | Inorganic ion transport | **B** | **A** |  | **B** | **A** |  |  |  |  |  |  |
|  | Nucleotide transport | **A** | **B** |  | **B** | **A** |  |  |  |  |  |  |
|  | Secondary metabolites | **B** | **A** |  | **B** | **A** |  |  |  |  |  |  |
| Note: Red and blue cells indicate significant positive or negative warming effects on gene abundances. MA and MI indicate macroaggregates and microaggregates. Different uppercase letters indicate differences between macroaggregates and microaggregates. | | | | | | | | | | | | |

| **Table S4.** Functional genes associated with ***cellular processes*** in soil aggregates over long-term warming. | | | | | | | | | | | | | |
| --- | --- | --- | --- | --- | --- | --- | --- | --- | --- | --- | --- | --- | --- |
| **Kingdom** | **Phylum** | **Aggregate** |  | **Cellular process** |  | **Cell wall** | **Defense mechanisms** | **Signal transduction** | **Translational chaperones** | **Cell division** | **Cell motility** | **Extracellular structures** | **Intracellular trafficking** |
| Archaea | Crenarchaeota | MA |  |  |  |  |  |  |  |  |  |  |  |
|  |  | MI |  |  |  |  |  |  |  |  |  |  |  |
|  | Euryarchaeota | MA |  |  |  |  |  |  |  |  |  |  |  |
|  |  | MI |  |  |  |  |  |  |  |  |  |  |  |
|  | Thaumarchaeota | MA |  |  |  |  |  |  |  |  |  |  |  |
|  |  | MI |  |  |  |  |  |  |  |  |  |  |  |
| Bacteria | Armatimonadetes | MA |  |  |  |  |  |  |  |  |  |  |  |
|  |  | MI |  |  |  |  |  |  |  |  |  |  |  |
|  | Bacteroidetes | MA |  |  |  |  |  |  |  |  |  |  |  |
|  |  | MI |  |  |  |  |  |  |  |  |  |  |  |
|  | Chloroflexi | MA |  |  |  |  |  |  |  |  |  |  |  |
|  |  | MI |  |  |  |  |  |  |  |  |  |  |  |
|  | Cyanobacteria | MA |  |  |  |  |  |  |  |  |  |  |  |
|  |  | MI |  |  |  |  |  |  |  |  |  |  |  |
|  | Deinococcus  Thermus | MA |  |  |  |  |  |  |  |  |  |  |  |
|  |  | MI |  |  |  |  |  |  |  |  |  |  |  |
|  | Firmicutes | MA |  |  |  |  |  |  |  |  |  |  |  |
|  |  | MI |  |  |  |  |  |  |  |  |  |  |  |
|  | Gemmatimonadetes | MA |  |  |  |  |  |  |  |  |  |  |  |
|  |  | MI |  |  |  |  |  |  |  |  |  |  |  |
|  | Nitrospirae | MA |  |  |  |  |  |  |  |  |  |  |  |
|  |  | MI |  |  |  |  |  |  |  |  |  |  |  |
|  | Spirochaetes | MA |  |  |  |  |  |  |  |  |  |  |  |
|  |  | MI |  |  |  |  |  |  |  |  |  |  |  |
|  | Verrucomicrobia | MA |  |  |  |  |  |  |  |  |  |  |  |
|  |  | MI |  |  |  |  |  |  |  |  |  |  |  |
| Fungi | Ascomycota | MA |  |  |  |  |  |  |  |  |  |  |  |
|  |  | MI |  |  |  |  |  |  |  |  |  |  |  |
|  | Basidiomycota | MA |  |  |  |  |  |  |  |  |  |  |  |
|  |  | MI |  |  |  |  |  |  |  |  |  |  |  |
|  | Chytridiomycota | MA |  |  |  |  |  |  |  |  |  |  |  |
|  |  | MI |  |  |  |  |  |  |  |  |  |  |  |
|  | Mucoromycota | MA |  |  |  |  |  |  |  |  |  |  |  |
|  |  | MI |  |  |  |  |  |  |  |  |  |  |  |
|  | Zoopagomycota | MA |  |  |  |  |  |  |  |  |  |  |  |
|  |  | MI |  |  |  |  |  |  |  |  |  |  |  |
| Note: Red and blue cells indicate significant positive or negative warming effects on gene abundances. MA and MI indicate macroaggregates and microaggregates. | | | | | | | | | | | | | |
|  | | | | | | | | | | | | | |
|  | | | | | | | | | | | | | |
| **Table S5.** Functional genes associated with ***metabolic pathways*** in aggregates over long-term warming. | | | | | | | | | | | | | |
| **Kingdom** | **Phylum** | **Aggregate** |  | **Metabolism** |  | **Amino acid transport** | **Carbohydrate transport** | **Energy production** | **Lipid transport** | **Coenzyme transport** | **Inorganic ion transport** | **Nucleotide transport** | **Secondary metabolites** |
| Archaea | Crenarchaeota | MA |  |  |  |  |  |  |  |  |  |  |  |
|  |  | MI |  |  |  |  |  |  |  |  |  |  |  |
|  | Euryarchaeota | MA |  |  |  |  |  |  |  |  |  |  |  |
|  |  | MI |  |  |  |  |  |  |  |  |  |  |  |
|  | Thaumarchaeota | MA |  | **B** |  |  |  |  |  |  |  |  |  |
|  |  | MI |  | **A** |  |  |  |  |  |  |  |  |  |
| Bacteria | Armatimonadetes | MA |  |  |  |  |  |  |  |  |  |  |  |
|  |  | MI |  |  |  |  |  |  |  |  |  |  |  |
|  | Bacteroidetes | MA |  |  |  |  |  |  |  |  |  |  |  |
|  |  | MI |  |  |  |  |  |  |  |  |  |  |  |
|  | Chloroflexi | MA |  |  |  |  |  |  |  |  |  |  |  |
|  |  | MI |  |  |  |  |  |  |  |  |  |  |  |
|  | Cyanobacteria | MA |  |  |  |  | **B** |  |  |  |  |  |  |
|  |  | MI |  |  |  |  | **A** |  |  |  |  |  |  |
|  | Deinococcus  Thermus | MA |  |  |  |  |  |  |  |  |  |  |  |
|  |  | MI |  |  |  |  |  |  |  |  |  |  |  |
|  | Firmicutes | MA |  |  |  |  |  |  |  |  |  |  |  |
|  |  | MI |  |  |  |  |  |  |  |  |  |  |  |
|  | Gemmatimonadetes | MA |  |  |  |  |  |  |  |  |  |  |  |
|  |  | MI |  |  |  |  |  |  |  |  |  |  |  |
|  | Nitrospirae | MA |  | **B** |  |  |  |  |  |  |  |  |  |
|  |  | MI |  | **A** |  |  |  |  |  |  |  |  |  |
|  | Spirochaetes | MA |  | **A** |  |  |  |  |  |  |  |  |  |
|  |  | MI |  | **B** |  |  |  |  |  |  |  |  |  |
|  | Verrucomicrobia | MA |  |  |  |  |  |  |  |  |  |  |  |
|  |  | MI |  |  |  |  |  |  |  |  |  |  |  |
| Fungi | Ascomycota | MA |  | **B** |  |  |  |  |  |  |  |  |  |
|  |  | MI |  | **A** |  |  |  |  |  |  |  |  |  |
|  | Basidiomycota | MA |  |  |  |  |  |  |  |  |  |  |  |
|  |  | MI |  |  |  |  |  |  |  |  |  |  |  |
|  | Chytridiomycota | MA |  |  |  |  |  |  |  |  |  |  |  |
|  |  | MI |  |  |  |  |  |  |  |  |  |  |  |
|  | Mucoromycota | MA |  |  |  |  |  |  |  |  |  |  |  |
|  |  | MI |  |  |  |  |  |  |  |  |  |  |  |
|  | Zoopagomycota | MA |  |  |  |  |  |  |  |  |  |  |  |
|  |  | MI |  |  |  |  |  |  |  |  |  |  |  |
| Note: Red and blue cells indicate significant positive or negative warming effects on gene abundances. MA and MI indicate macroaggregates and microaggregates. Different uppercase letters indicate differences between macroaggregates and microaggregates. | | | | | | | | | | | | | |


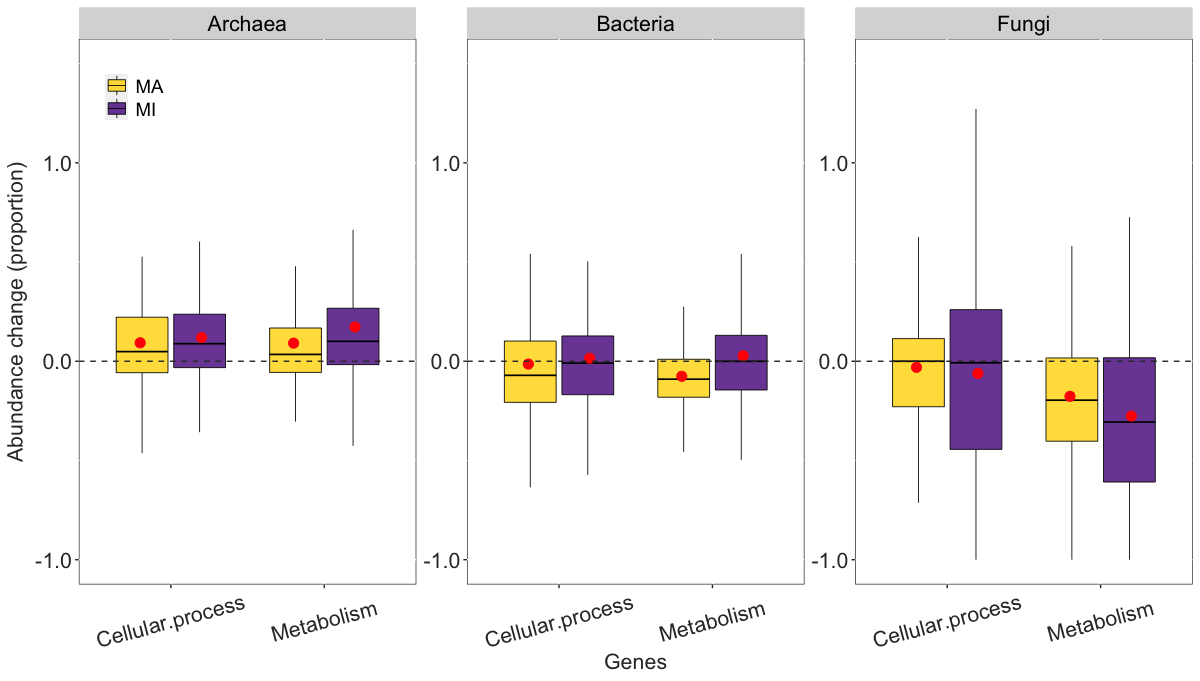


P_agg_=0.33, P_class_<0.01

P_kingdom_<0.01, P_agg_xP_kingdom_<0.01

**#**

*

**

***

*

**

***

***

***

*

**Figure S1.** Changes of microbial functional genes in different aggregates under long-term warming. MA and MI are macro- and micro- aggregates (250-2000; <250 µm). Black symbols indicate significant warming effects ((heated-control)/control), while red symbols indicate significant differences between MA and MI (#, *, **, *** at P < 0.10, 0.05, 0.01, and 0.001).

*

***

#

**
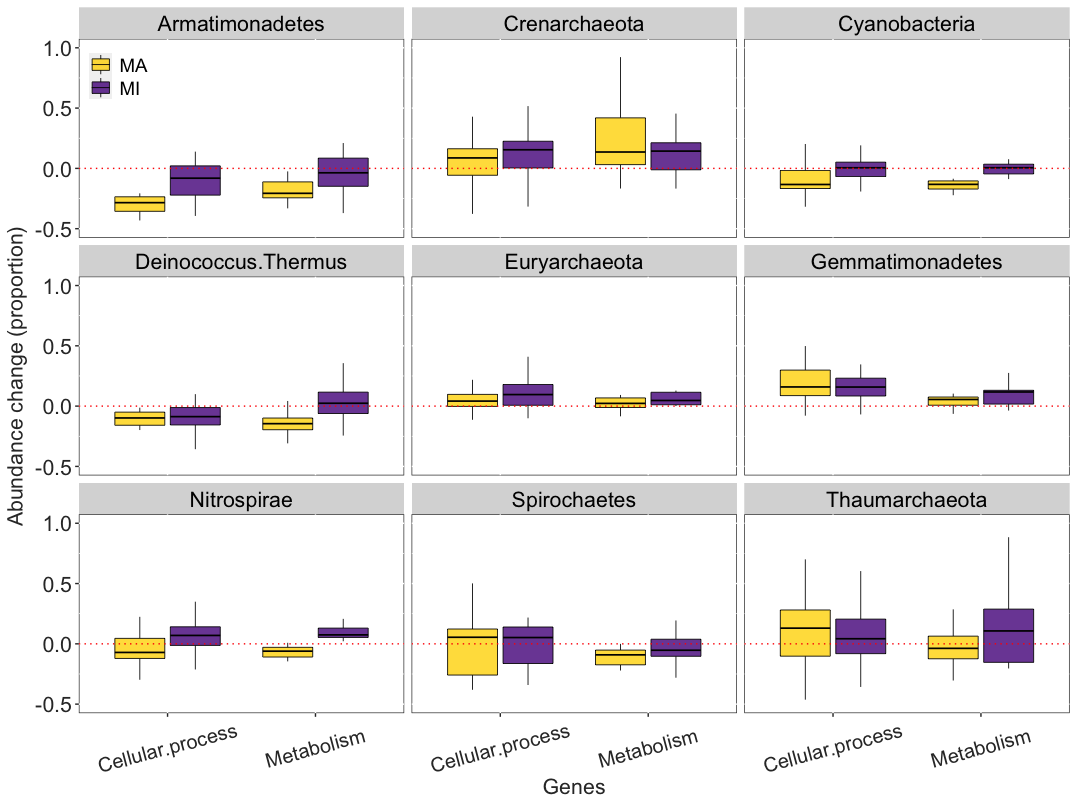
**

**

**

*

**

**

***

**

**

***

*

*

*

**#**

*

**#**

**

***

*

*

**#**

***

**#**

**#**

***

***

**Figure S2.** Changes in relative abundances of archaeal and bacterial genes in different aggregates under long-term warming. MA and MI are macro- and micro- aggregates (250-2000; <250 µm). Black symbols indicate significant warming effects ((heated-control)/control), while red symbols indicate significant differences between MA and MI (#, *, **, *** at P < 0.10, 0.05, 0.01, and 0.001).

**
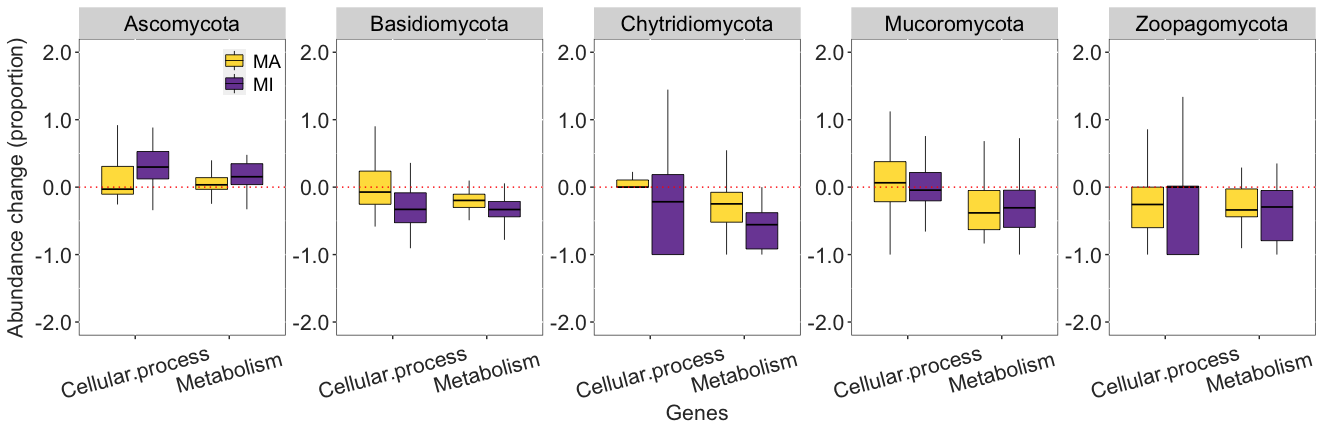
**

*

***

**

**#**

**#**

**#**

**

***

**

**

**

*

**#**

**Figure S3.** Changes in relative abundances of fungal genes in different aggregates under long-term warming. MA and MI are macro- and micro- aggregates (250-2000; <250 µm). Black symbols indicate significant warming effects ((heated-control)/control), while red symbols indicate significant differences between MA and MI (#, *, **, *** at P < 0.10, 0.05, 0.01, and 0.001).

**
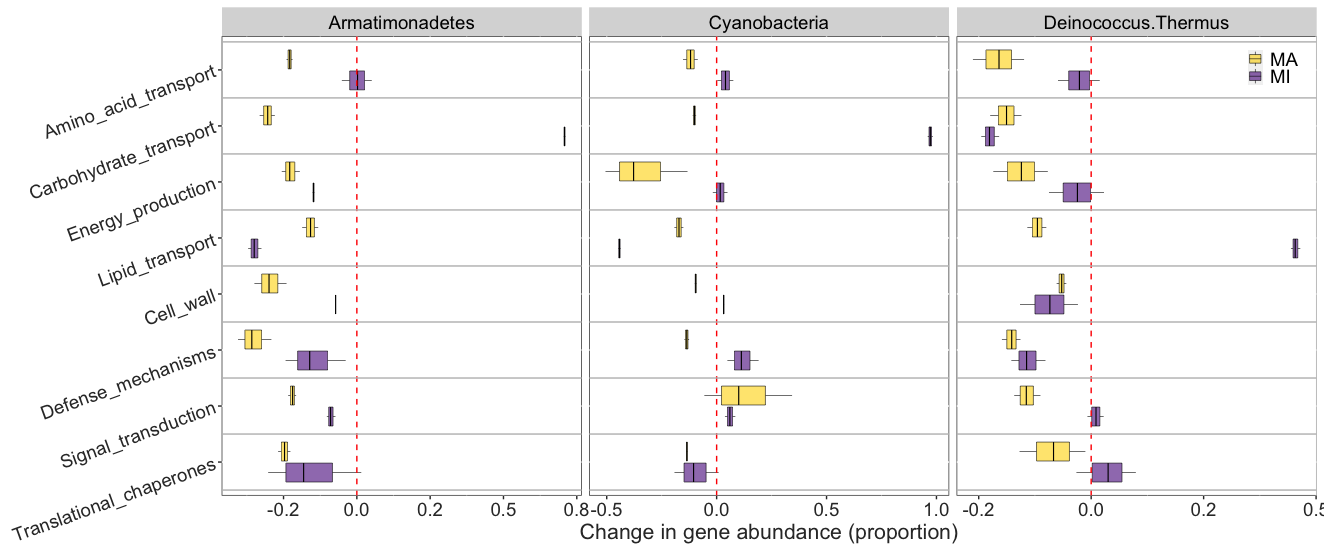
**

**

**

***

**

**#**

***

**

***

**

**

#

*

***

*

**

*

*

***

*

**

**

*

*

***

**

***

**

**

**

***

*

**

**

**

**

**
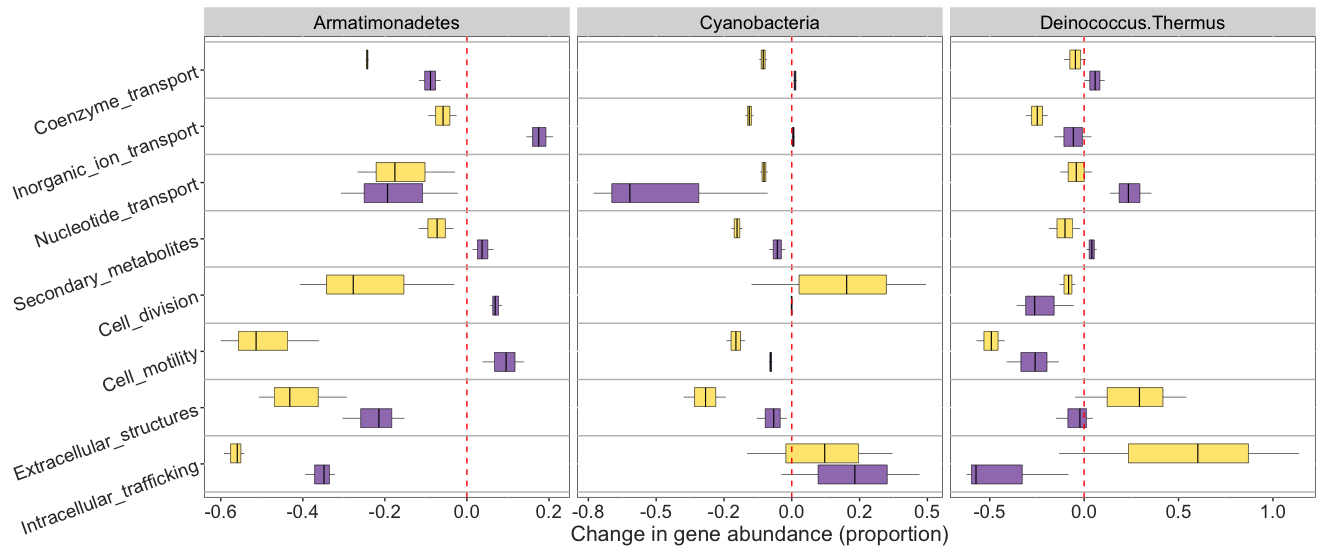
**

**

**#**

**

**

**

**#**

**

**

*

**#**

***

*

*

**#**

*

**#**

*

*

*

***

**

*

*

**#**

**

**#**

**Figure S4.** Changes in relative abundances of bacterial genes in different aggregates under long-term warming. MA and MI are macro- and micro- aggregates (250-2000; <250 µm). Black symbols indicate significant warming effects ((heated-control)/control), while red symbols indicate significant differences between MA and MI (#, *, **, *** at P < 0.10, 0.05, 0.01, and 0.001).

**
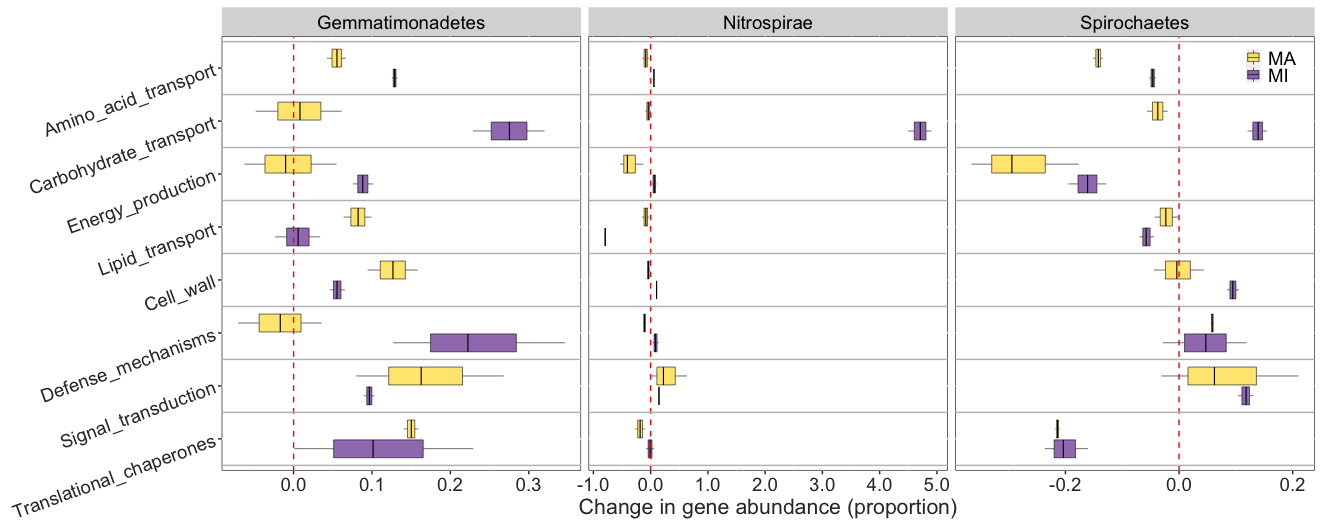
**

**#**

**

**#**

***

***

*

**#**

***

**#**

**

**

**

*

*

*

*

***

**

***

**

*

***

*

**

**

*

*

**

**#**

**#**

**

***

**
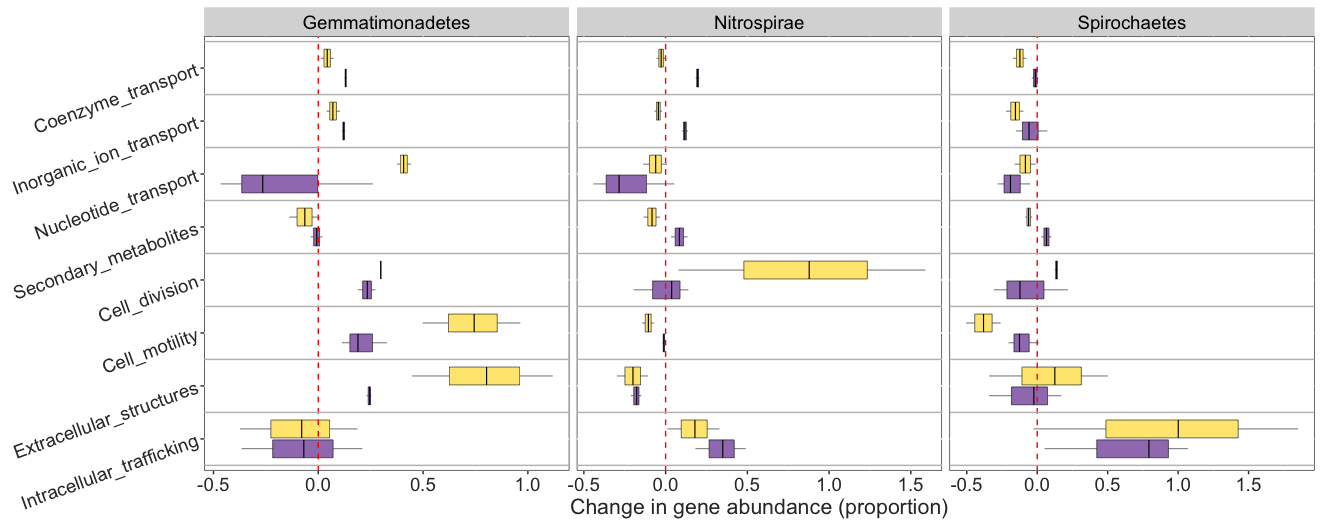
**

**

**#**

**

**#**

**#**

*

**#**

*

**#**

***

**

*

**

*

***

*

**#**

*

***

*

*

*

**#**

**

*

**Figure S5.** Changes in relative abundances of bacterial genes in different aggregates under long-term warming. MA and MI are macro- and micro- aggregates (250-2000; <250 µm). Black symbols indicate significant warming effects ((heated-control)/control) (#, *, **, *** at P < 0.10, 0.05, 0.01, and 0.001).

**
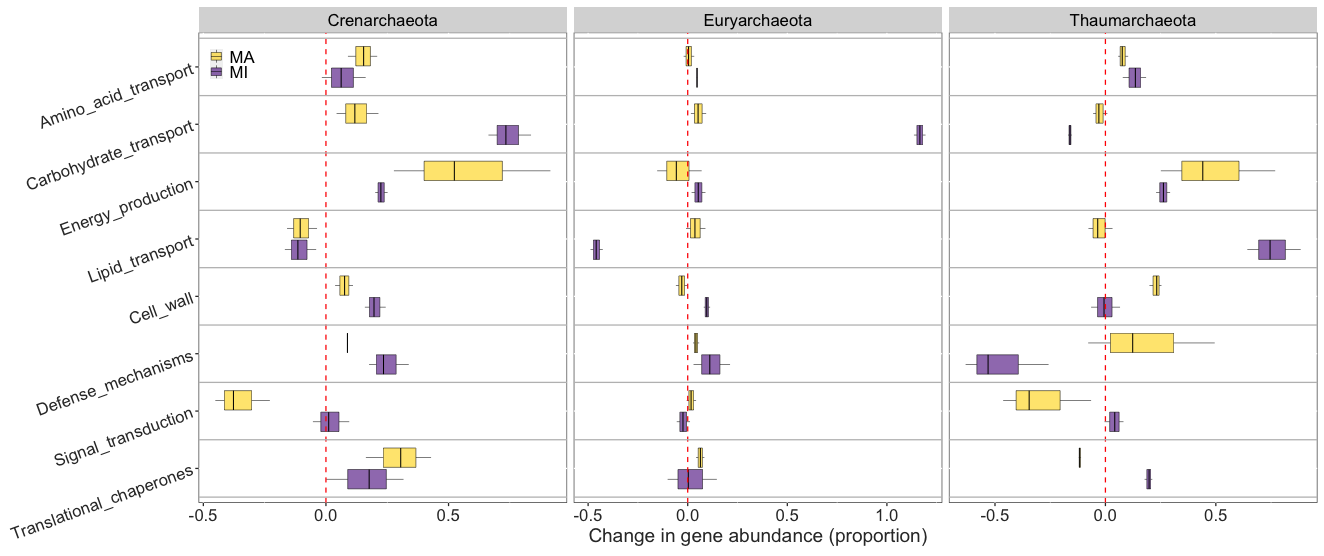
**

*

*

***

**

**#**

**

**

*

**

***

***

***

**

*

***

*

*

**

**

**#**

**#**

**#**

*

**#**

*

***

*

**#**

**
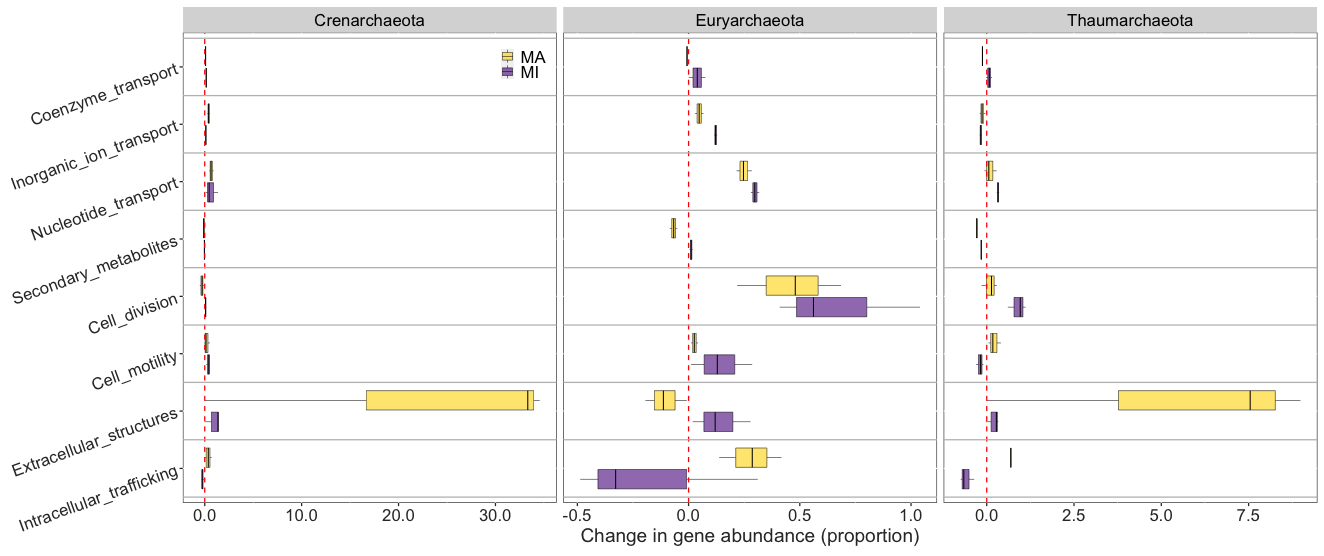
**

*

*

**

*

*

**#**

**#**

*

*

**

**

**

*

**#**

**#**

*

***

*

**

***

**

*

**#**

*

***

**Figure S6.** Changes in relative abundances of archaeal genes in different aggregates under long-term warming. MA and MI are macro- and micro- aggregates (250-2000; <250 µm). Black symbols indicate significant warming effects ((heated-control)/control) (#, *, **, *** at P < 0.10, 0.05, 0.01, and 0.001).

**
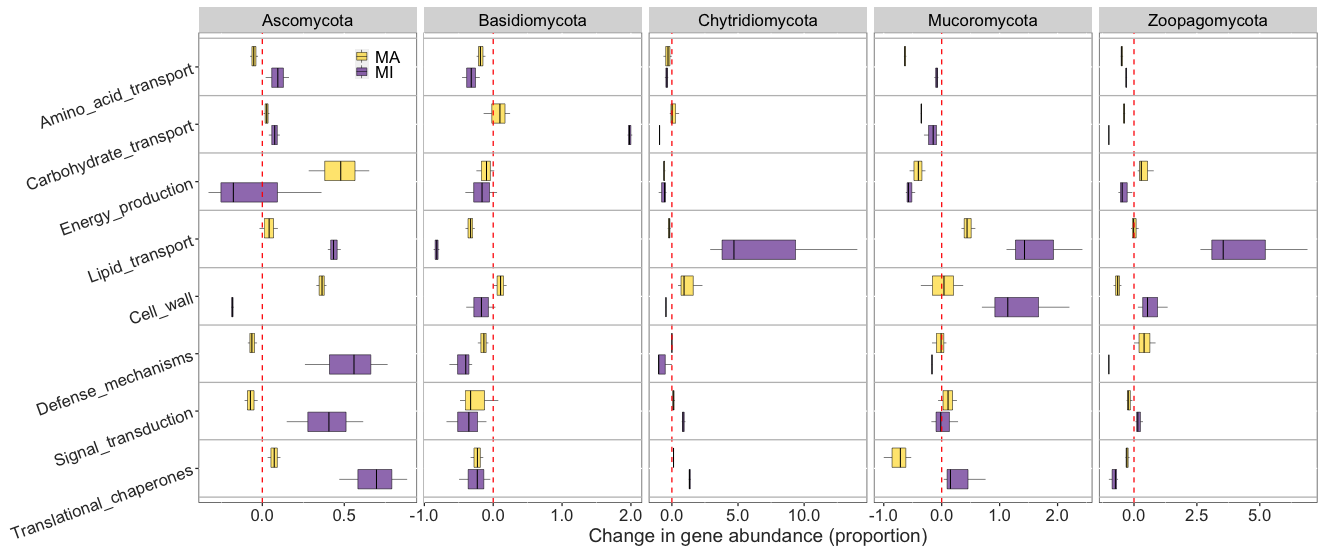
**

**#**

**#**

*

**

***

**

**#**

**#**

**#**

**#**

**#**

*

*

*

***

***

*

**#**

*

*

*

***

*

**

**#**

***

**

**

**

***

*

***

*

**

*

*

***

**#**

*

**

**

**

**

*

*

**#**

*

*

**#**

**
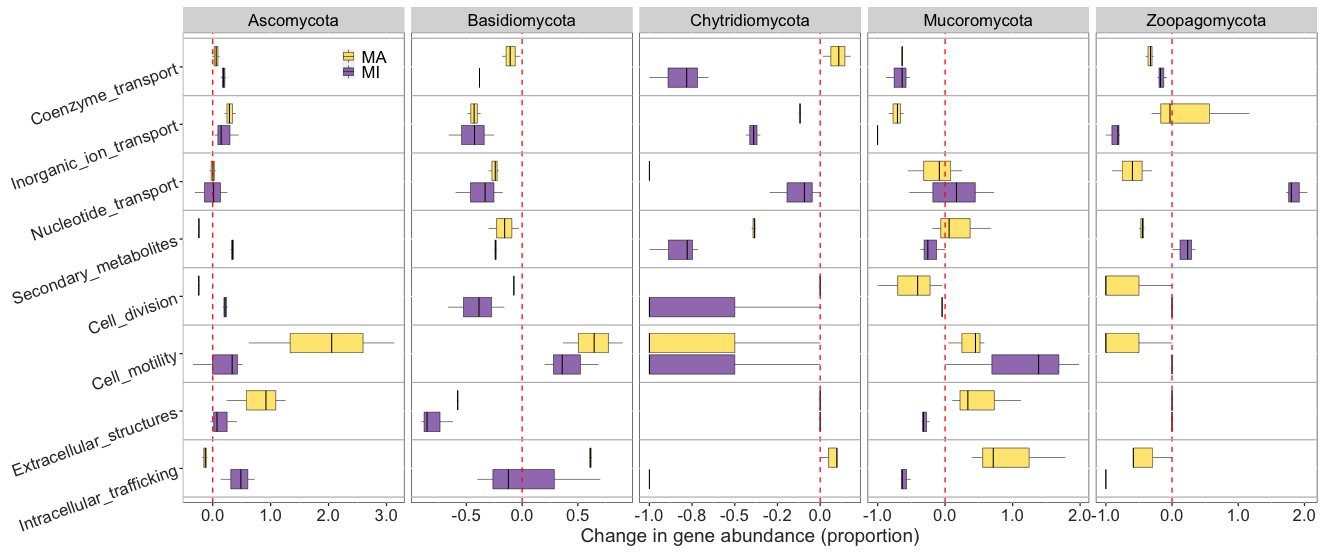
**

***

*

**

**

**

*

**

*

**#**

**

**#**

**

**

**#**

*

*

***

**

**

*

**#**

**#**

*

**#**

***

**

*

*

**#**

***

***

**#**

*

**#**

***

*

***

*

**

**

***

*

**#**

**Figure S7.** Changes in relative abundances of fungal genes in different aggregates under long-term warming. MA and MI are macro- and micro- aggregates (250-2000; <250 µm). Black symbols indicate significant warming effects ((heated-control)/control) (#, *, **, *** at P < 0.10, 0.05, 0.01, and 0.001).

**
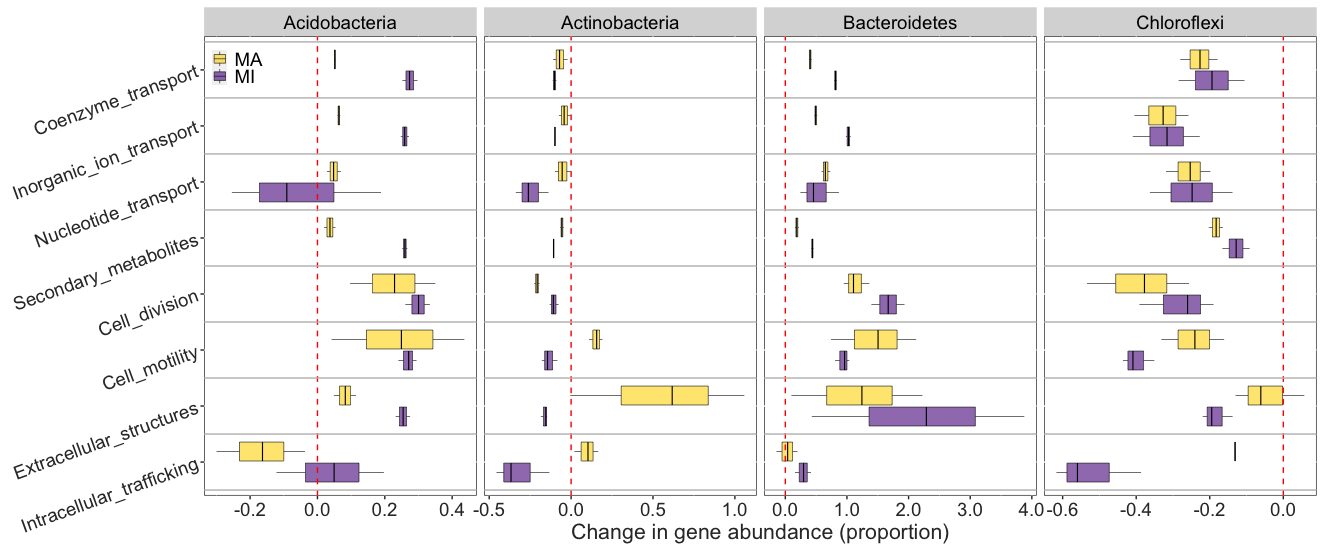
**

*

*

*

*

*

*

*

**

*

*

*

**

*

***

*

***

**

***

***

**#**

**

***

**

*

**

*

**

**#**

**

**#**

***

**

*

***

***

***

*

*

**

*

*

**

**#**

***

**

***

***

***

***

**

***

*

***

***

*

**

**#**

**

**

*

***

**
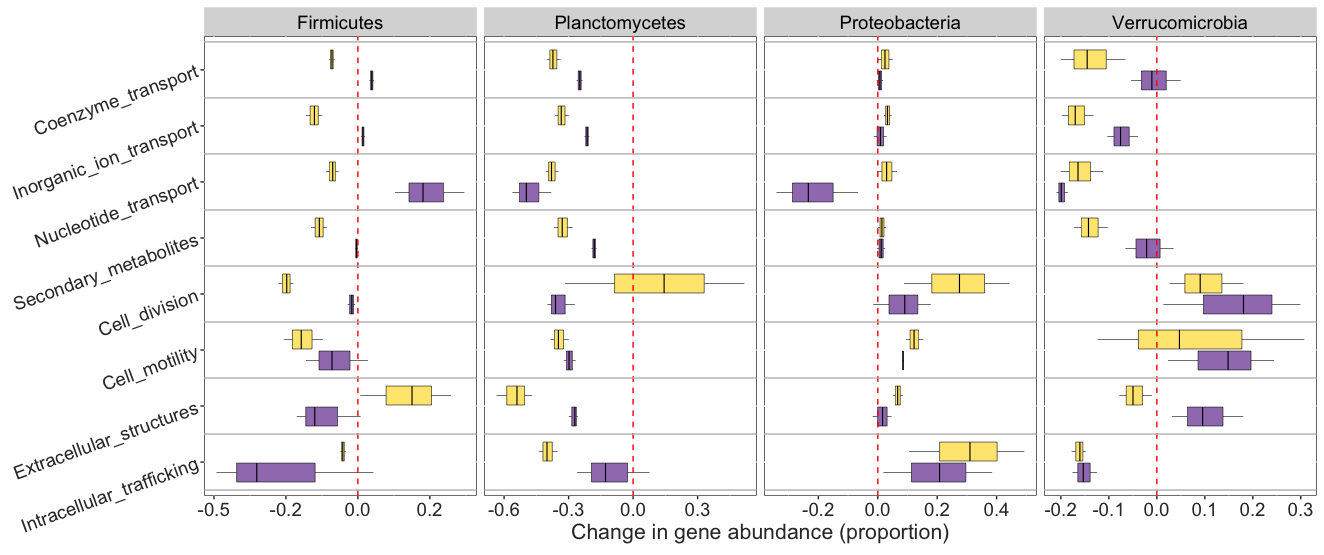
**

**

**

**

***

**

*

**

**

**

*

**

**

**

**

**#**

*

**

**

**

*

*

*

**#**

*

**

**#**

*

*

**#**

*

*

**

*

*

**

**

**#**

*

*

***

**#**

**#**

***

*

*

**Figure S8.** Changes in relative abundances of bacterial genes in different aggregates under long-term warming. MA and MI are macro- and micro- aggregates (250-2000; <250 µm). Black symbols indicate significant warming effects ((heated-control)/control), while red symbols indicate significant differences between MA and MI (#, *, **, *** at P < 0.10, 0.05, 0.01, and 0.001).

**Figure S9.** Abundances of degradation genes associated with different substrates from different enzyme classes as mediated by aggregate size in response to long-term warming (different letters indicate differences between macroaggregates and microaggregates in either control (grey) or warmed (red) plots). *, **, and *** indicate significant difference between Control vs heated at P < 0.05, 0.01, and 0.001 from one-way ANOVA. ΑA, auxiliary activities; CE, carbohydrate esterases; GH, glycosidase hydrolases; PL, polysaccharide lyases.


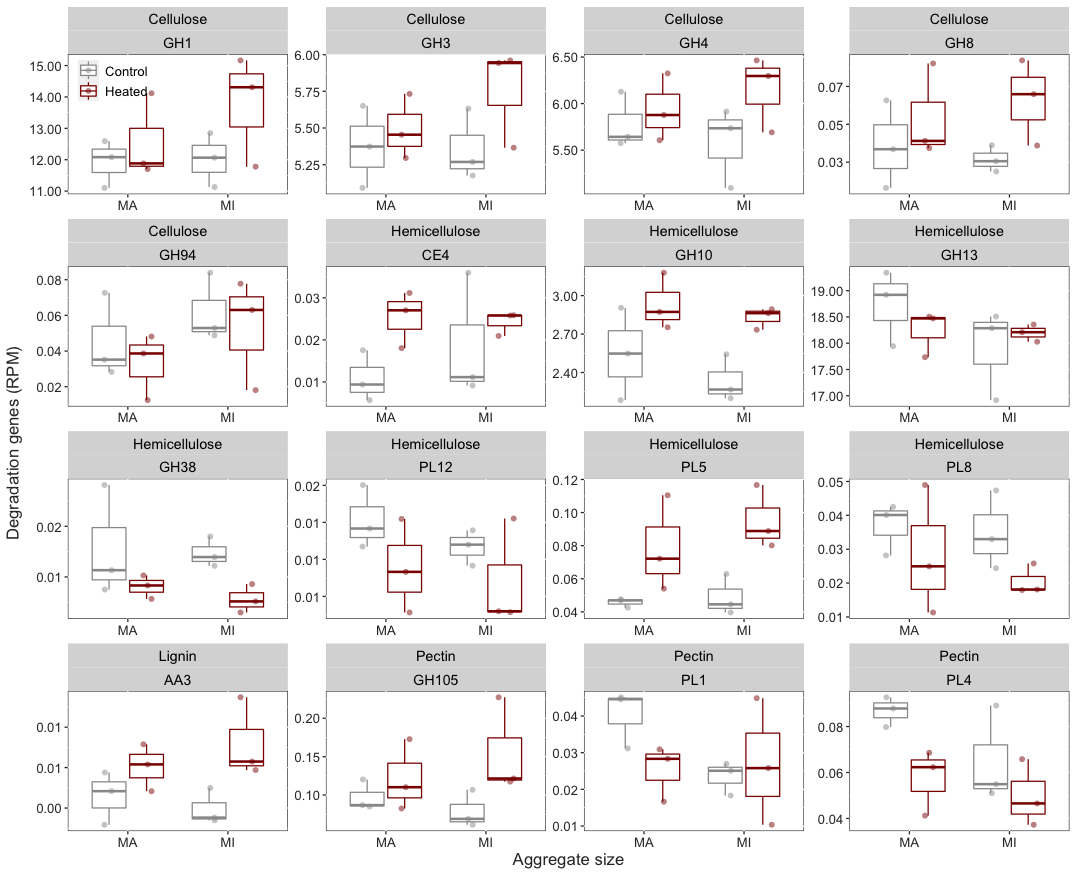


*

**

**

***

A

**

B

*

*

*

A

***

*

A

B

B

*

***

***

*

***

*

A

B

*

*

***

B

A

**

*

*


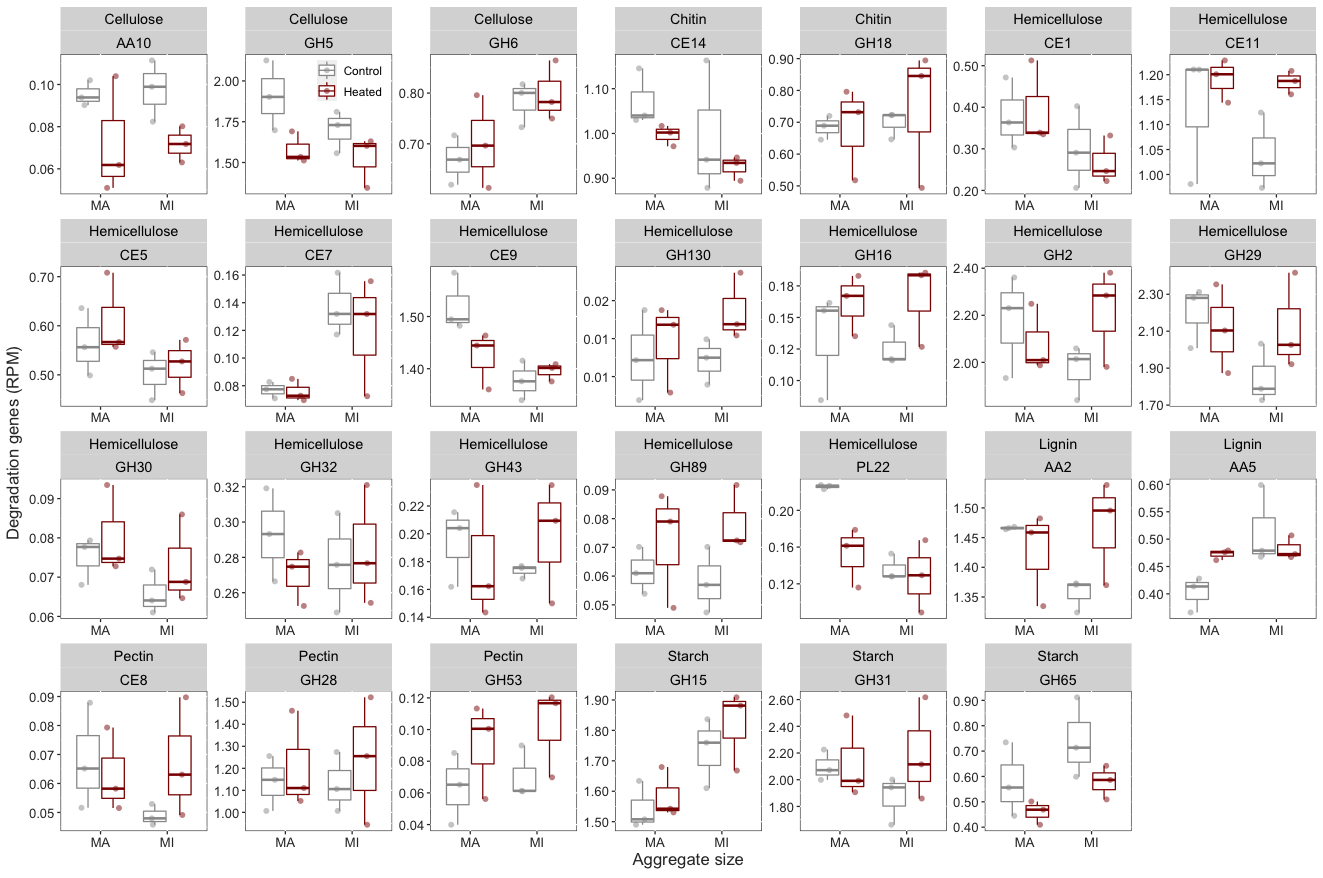


A

B

A

B

**Figure S10.** Abundances of degradation genes associated with different substrates from different enzyme classes as mediated by aggregate size in response to long-term warming (different letters indicate differences between macroaggregates and microaggregates in control (grey) plots). ΑA, auxiliary activities; CE, carbohydrate esterases; GH, glycosidase hydrolases; PL, polysaccharide lyases.

.


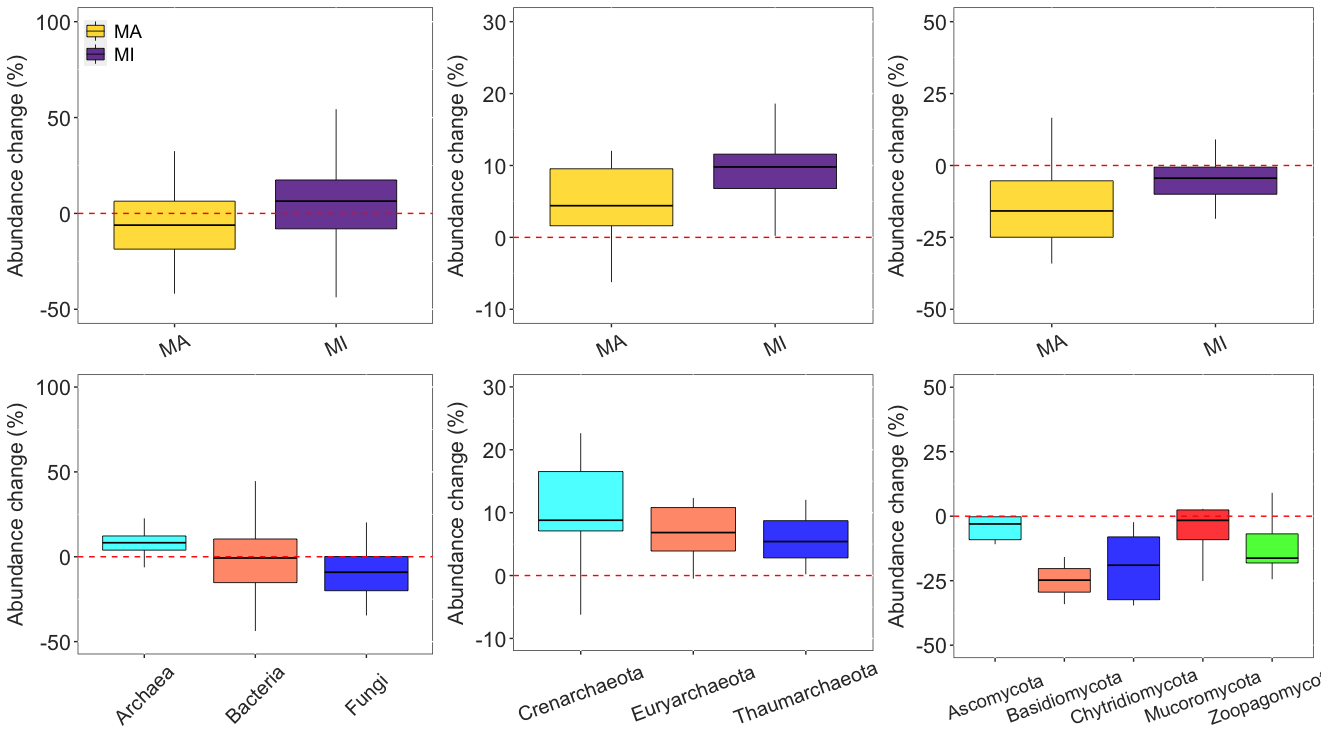


**

**

B

B

A

AB

A

B

B

A

***

**

*

C

**

#

#

*

#

#

A

**Figure S11.** Changes of relative abundances in across all kingdoms, archaea, and fungi as mediated by aggregate size in response to long-term warming. MA and MI are macro- and micro- aggregates (250-2000; <250 µm). Red symbols indicate significant warming effects ((heated-control)/control × 100%) (#, *, **, *** at P < 0.10, 0.05, 0.01, and 0.001). Different uppercase letters indicate differences between kingdoms or phyla.

**Figure S12.** Changes of archaeal and fungal relative abundances as mediated by aggregate size in response to long-term warming. MA and MI are macro- and micro- aggregates (250-2000; <250 µm). Black symbols indicate significant warming effects ((heated-control)/control × 100%), while red symbols indicate significant differences between MA and MI (#, *, **, *** at P < 0.10, 0.05, 0.01, and 0.001).


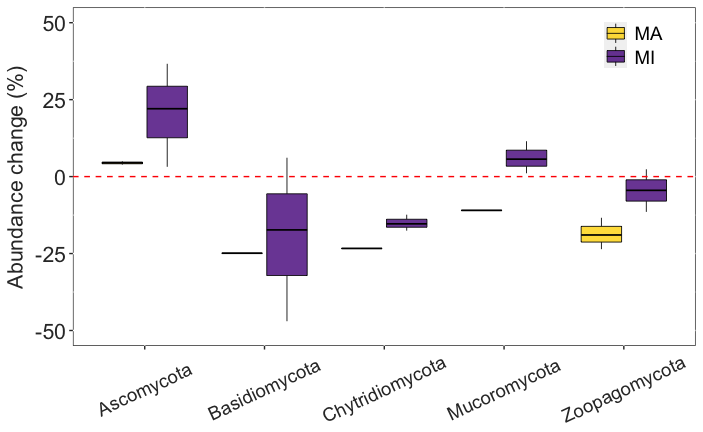

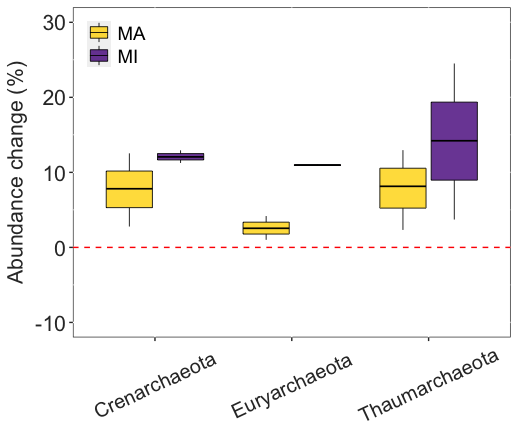


P_a_<0.01, P_p_=0.27, P_a×p_=0.76

P_a_<0.01, P_p_<0.01, P_a×p_=0.83

*

**

*

#

#

#

*

*

**

#


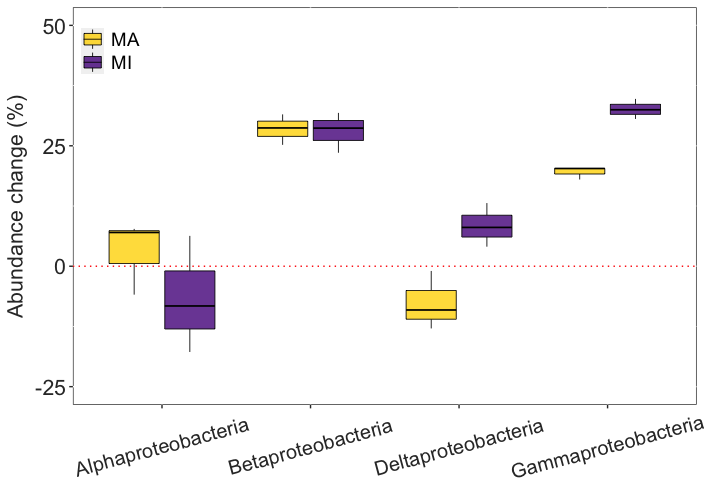


*

***

***

*

***

***

*

***

***


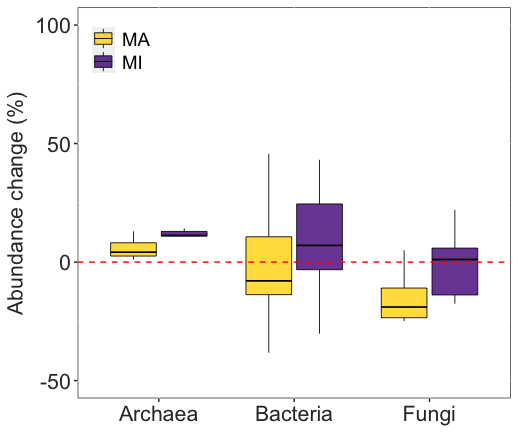


P_a_=0.04, P_k_<0.01, P_a×k_=0.86

**

**

**

*

**Figure S13.** Changes of relative abundances at the kingdom level and in classes of Proteobacteria as mediated by aggregate size in response to long-term warming. MA and MI are macro- and micro- aggregates (250-2000; <250 µm). Black symbols indicate significant warming effects ((heated-control)/control × 100%), while red symbols indicate significant differences between MA and MI (*, **, *** at P < 0.05, 0.01, and 0.001).


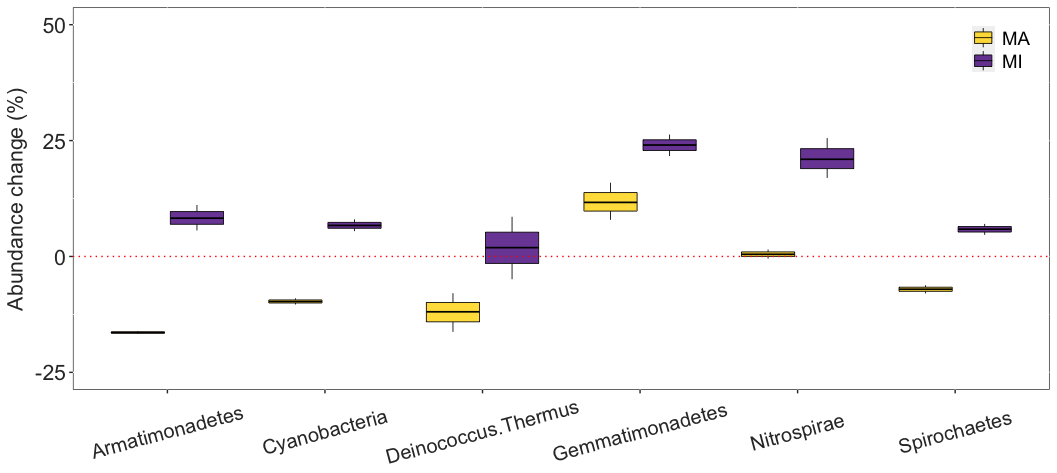


**Figure S14.** Changes of bacterial relative abundances as mediated by aggregate size in response to long-term warming. MA and MI are macro- and micro- aggregates (250-2000; <250 µm). Black symbols indicate significant warming effects ((heated-control)/control × 100%), while red symbols indicate significant differences between MA and MI (*, **, *** at P < 0.05, 0.01, and 0.001).

P_a_<0.01, P_p_<0.01, P_a×p_<0.01

*

*

*

*

*

**

**

**

**

**

**

*

***

**References**

1. Bredon M, Dittmer J, Noël C, Moumen B, Bouchon D. Lignocellulose degradation at the holobiont level: teamwork in a keystone soil invertebrate. *Microbiome* 2018; **6**: 162.

2. Cardenas E, Orellana LH, Konstantinidis KT, Mohn WW. Effects of timber harvesting on the genetic potential for carbon and nitrogen cycling in five North American forest ecozones. *Sci Rep* 2018; **8**: 3142.

3. Cardenas E, Kranabetter JM, Hope G, Maas KR, Hallam S, Mohn WW. Forest harvesting reduces the soil metagenomic potential for biomass decomposition. *ISME J* 2015; **9**: 2465–2476.

4. Lemos LN, Pereira RV, Quaggio RB, Martins LF, Moura LMS, da Silva AR, et al. Genome-centric analysis of a thermophilic and cellulolytic bacterial consortium derived from composting. *Front Microbiol* 2017; **8**: 00644.

5. López-Mondéjar R, Tláskal V, Větrovský T, Štursová M, Toscan R, Nunes da Rocha U, et al. Metagenomics and stable isotope probing reveal the complementary contribution of fungal and bacterial communities in the recycling of dead biomass in forest soil. *Soil Biol Biochem* 2020; **148**: 107875.

6. Pold G, Billings AF, Blanchard JL, Burkhardt DB, Frey SD, Melillo JM, et al. Long-term warming alters carbohydrate degradation potential in temperate forest soils. *Appl Environ Microbiol* 2016; **82**: 6518–6530.
